# Phytotoxic Mechanisms of Polystyrene Microplastics in *Myriophyllum spicatum* Under Saline Conditions: Insights from Physiology, Transcriptomics, and Phyllosphere Microbiota

**DOI:** 10.3390/toxics14050416

**Published:** 2026-05-10

**Authors:** Junyu Xuan, Jinquan Wan, Lanhui Wen, Yan Wang, Ji Shiming

**Affiliations:** 1 Department of Environment and Energy, South China University of Technology, Guangzhou 510006, China; 202320146546@mail.scut.edu.cn (J.X.); 202421047604@mail.scut.edu.cn (L.W.); yanwang@scut.edu.cn (Y.W.); 2 Guangdong Shunkong Zihua Technology Co., Ltd., Foshan 528300, China; jsmsunny@163.com

**Keywords:** microplastics, *Myriophyllum spicatum*, ecotoxicity, oxidative stress, phyllosphere microbiota, nutrient cycling

## Abstract

Microplastics are emerging contaminants widely present in aquatic environments, yet their toxic effects on submerged plants and associated microbial communities under saline conditions remain unclear. In this study, *Myriophyllum spicatum* was exposed to polystyrene (PS) microplastics (0, 10, 30, 60, and 100 mg·L^−1^) under 0.5% salinity. We investigated plant growth, physiological responses, nitrogen and phosphorus removal, transcriptomic changes, and phyllosphere microbial communities. Results showed a concentration-dependent response, with low-dose stimulation and high-dose inhibition. At 30 mg·L^−1^, PS promoted growth, maintained membrane integrity and photosynthetic pigment levels, and enhanced nutrient removal. In contrast, 100 mg·L^−1^ PS caused membrane damage, photosynthetic inhibition, oxidative stress, and reduced nutrient uptake, indicating clear toxic effects. Transcriptomic analysis revealed that high PS significantly affected genes related to photosynthesis, antioxidant defense, energy metabolism, and nutrient transport. Microplastics promoted biofilm formation on leaf surfaces but did not significantly alter overall microbial community structure or diversity, instead shifting functionally related taxa associated with plant oxidative responses and nutrient removal. These findings demonstrate that PS microplastics exert phytotoxic effects under saline conditions by disrupting physiological processes and are associated with shifts in functional microbial groups, with potential implications for aquatic ecosystem health.

## 1. Introduction

Microplastics (MPs) are generally defined as plastic particles smaller than 5 mm in size and have become a widely distributed emerging pollutant in aquatic environments [1]. Due to their high environmental persistence, small particle size, and large specific surface area, microplastics can interact with aquatic organisms and environmental processes through multiple pathways [2,3]. Increasing evidence indicates that microplastics not only exert direct effects on individual organisms but may also influence aquatic ecosystems by altering microbial community structures and biogeochemical cycling processes [4,5]. More importantly, microplastics have been increasingly recognized as potential toxic agents, capable of inducing physiological disturbances such as oxidative stress, membrane damage, and metabolic disruption in aquatic organisms. Currently, studies on the ecological effects of microplastics have mainly focused on marine animals and plankton [6,7], whereas research on aquatic plants, particularly submerged macrophytes, remains relatively limited.

At the same time, salinity variation has emerged as an important environmental stressor affecting aquatic ecosystems in recent years [8]. In estuarine and coastal waters, seawater intrusion and climate change frequently lead to increased salinity, thereby significantly altering the physicochemical properties of water bodies and affecting aquatic community structures [9,10]. Elevated salinity not only imposes osmotic stress on plants but may also modify microbial community composition and function [11]. In addition, estuarine regions are often important sinks for microplastic pollution, and studies have shown that salinity changes and microplastic contamination frequently co-occur in these environments [12]. Therefore, under saline conditions, the ecological effects of microplastics may differ from those in freshwater systems and result in more complex environmental stress. However, research on the combined toxic effects of microplastics and salinity on aquatic plants and their associated microbial communities remains limited, particularly regarding their interactive impacts on plant physiological processes and ecosystem functions.

Submerged macrophytes are key components of freshwater and estuarine ecosystems. They not only contribute to primary productivity through photosynthesis but also stabilize sediment structure, provide habitats for aquatic organisms, and play a crucial role in maintaining ecosystem stability [13,14]. Furthermore, in ecological restoration and water purification systems, submerged plants can effectively remove nutrients such as nitrogen and phosphorus through direct uptake and synergistic interactions with microorganisms, thereby improving water quality [15]. Under single salinity stress, previous studies have mainly focused on its effects on osmotic regulation and the photosynthetic system. For example, *Vallisneria natans* exhibited significant reductions in chlorophyll content and photosynthetic efficiency under elevated salinity, accompanied by a certain degree of impairment in membrane stability [16]. Similarly, *Ceratophyllum demersum* showed damage to the photosynthetic system and inhibited growth under salinity stress [17]. Under single microplastic stress, studies have indicated that microplastics may influence the growth and physiological status of aquatic plants through mechanisms such as surface attachment, alteration of light conditions, and induction of oxidative stress [18,19]. The concentration-dependent effect of microplastics is also an important factor influencing plant responses. For instance, exposure to high concentrations of polystyrene microplastics (PS) led to reduced photosynthetic pigment content and inhibited growth in *Elodea nuttallii* [20].

Although previous studies have separately examined the effects of salinity and microplastics on plants, systematic analyses of their interactive effects on the physiological processes and molecular response mechanisms of submerged macrophytes under saline conditions remain limited. Particularly in estuarine environments, where salinity and microplastic pollution often coexist, such combined stressors may produce more complex ecological effects than single factors alone. Under multiple environmental stress conditions, plant responses are typically more complex. For example, the combined effects of salinity and heavy metals can cause significant growth inhibition and oxidative damage in *Suaeda salsa* [21]. When microplastics coexist with heavy metals, they can adsorb metal ions and alter their bioavailability, thereby affecting pollutant uptake and physiological responses in plants [22]. However, the response mechanisms of submerged macrophytes under combined salinity and microplastic stress, particularly their interactions with phyllosphere microbial communities, remain a significant knowledge gap.

The phyllosphere of aquatic plants typically harbors abundant microbial communities, which are involved in various ecological processes such as organic matter degradation and nutrient cycling [23,24]. Owing to their large specific surface area and unique physicochemical properties, microplastics can serve as potential substrates for microbial attachment and colonization. These changes in microbial communities may further influence plant stress responses by modulating microbially mediated nutrient cycling and redox-related processes, thereby indirectly affecting plant tolerance to microplastic-induced stress [25]. However, the effects of microplastics on phyllosphere microbial communities of submerged plants and their potential links to plant physiological responses remain unclear.

*Myriophyllum spicatum* is a widely distributed submerged macrophyte characterized by rapid growth, strong environmental adaptability, and high nutrient uptake capacity and is therefore widely used in aquatic ecological restoration and eutrophication control [26]. Due to its sensitivity to environmental changes, *M. spicatum* is also commonly used as a model plant for studying stress responses in aquatic plants [27]. Therefore, investigating the effects of microplastics on the physiological processes and ecological functions of *M. spicatum* under saline conditions is of great significance for assessing the potential ecological risks of microplastics in aquatic ecosystems.

Based on this, *Myriophyllum spicatum* was selected as the model species in this study, and different concentrations of polystyrene microplastics (PS) were applied under a saline background. We hypothesize that PS exerts concentration-dependent effects on *M. spicatum*, influencing plant growth, photosynthetic performance, oxidative stress, and nutrient removal capacity. Furthermore, these physiological responses are expected to be associated with changes in gene expression and may be linked to shifts in specific microbial taxa rather than large-scale alterations in overall community structure. To test these hypotheses, this study aims to (1) evaluate the effects of different PS concentrations on plant growth and physiological responses; (2) elucidate the molecular regulatory mechanisms underlying plant responses to PS stress using transcriptomic analysis; and (3) investigate changes in phyllosphere microbial communities and their potential associations with plant physiological functions.

## 2. Materials and Methods

### 2.1. Chemicals and Plant Materials

Polystyrene (PS) powder with a nominal particle size of 3 μm was obtained from Dongguan Plastic Raw Material Development Center (Dongguan, China). Sea salt was purchased from Qinhuangdao Leyu Aquaculture Technology Co., Ltd. (Qinhuangdao, China). The physicochemical properties of the PS powder (Figures S1–S3) and the detailed composition of the sea salt (Table S1) are provided in the Supplementary Materials.

Mature *Myriophyllum spicatum* plants were purchased from Zhongshan Horticulture Company (Zhongshan, China). To remove snails attached to the plant surface, the plants were immersed in a 3 g·L^−1^ CuSO_4_ solution for 10 min, followed by rinsing with deionized water and soaking in clean water for 1 h. The plants were then thoroughly washed and pre-cultured in Hoagland nutrient solution (Table S2) for one week to ensure uniform initial conditions. Pre-cultivation was conducted under controlled laboratory conditions simulating natural environments, with a 12 h light/12 h dark photoperiod, a temperature of 25 ± 2 °C, and a light intensity of 4000 lux.

### 2.2. Experimental Design

To simulate eutrophic estuarine water conditions, experimental water was collected from a highly eutrophic river in China (22°44′26″ N, 113°44′53″ E) and filtered through a 0.45 μm membrane before use. The initial nutrient concentrations are provided in Table S3, with ammonium nitrogen (NH_4_^+^-N) ranging from 9.12 to 9.23 mg·L^−1^ and total phosphorus (TP) from 1.20 to 1.23 mg·L^−1^. To ensure consistent initial nutrient conditions across treatments, NH_4_^+^-N and TP concentrations were adjusted to 10 mg·L^−1^ and 2 mg·L^−1^ using NH_4_Cl and KH_2_PO_4_, respectively. The experiment was conducted in cylindrical plexiglass containers (height 400 mm, diameter 150 mm, wall thickness 3 mm). Each container was filled with 5 L of prepared water and layered with 30 mm of quartz sand substrate (particle size 2–7 mm; Aladdin Biochemical Technology Co., Ltd., Shanghai, China). Prior to planting, the leaves of *Myriophyllum spicatum* were gently rinsed with deionized water to remove pre-existing epiphytic biofilms.

Based on previous studies, 0.5% salinity falls within the suitable range for normal growth of *M. spicatum* [28]; therefore, it was selected as the background salinity to simulate estuarine conditions. Based on the ionic composition of the solution, the corresponding osmotic potential (Text S1) was estimated to be approximately −0.33 MPa, indicating a mild saline condition with relatively limited osmotic stress on the plant. Based on previous ecotoxicological studies, five PS concentration gradients (0, 10, 30, 60, and 100 mg·L^−1^) were established to investigate concentration-dependent responses [29]. These concentrations are commonly applied in laboratory studies to enable a clearer assessment of dose-dependent effects under controlled conditions. Although environmental concentrations are typically lower, microplastic levels in estuarine systems may vary due to spatial heterogeneity and local accumulation processes [30,31]. Each treatment included three biological replicates. The experiment lasted for 28 days under the same conditions as the pre-cultivation stage. Representative growth status of *M. spicatum* at day 14 is shown in Figure S4. Water quality parameters were monitored every 4 days, and distilled water was added daily to compensate for evaporation losses. At the end of the experiment, plant tissues and leaf-associated biofilm samples were collected for subsequent physiological measurements, transcriptomic analysis, and microbial community analysis.

### 2.3. Plant Growth and Chlorophyll Measurement

Plant fresh weight and shoot height were recorded at the beginning and end of the experiment to evaluate plant growth. Electrolyte leakage (EL) of leaves was measured to assess cell membrane integrity, following the method of Gulen and Eris [32] with slight modifications (Text S2). In addition, leaf tissues from different treatments were observed using an optical microscope (Olympus BX53, Olympus Corporation, Tokyo, Japan) at a magnification of 40× to examine cellular morphology. Chlorophyll (chl) content was used as an indicator of photosynthetic activity. Approximately 0.2 g of fresh leaf tissue was collected, washed, and air-dried, then immersed in 10 mL of 96% ethanol for pigment extraction in the dark for 24 h. The absorbance of the extract was measured at 645 and 663 nm using a UV-Vis spectrophotometer (Evolution 201, Thermo Fisher Scientific, Waltham, MA, USA). Total chlorophyll content was calculated using the following equations:

chl a = 12.7 × D663 − 2.69 × D645(1)

chl b = 22.9 × D645 − 2.69 × D663(2)

Total chl = chl a + chl b(3)

### 2.4. Determination of MDA Content and Antioxidant Enzyme Activities

On day 28 of the experiment, approximately 0.1 g of fresh leaf tissue was collected from each treatment. Under ice-bath conditions, the samples were thoroughly homogenized using the extraction buffer provided in the corresponding assay kits at a ratio of tissue mass (g) to extraction volume (mL) of 1:10. The homogenate was centrifuged at 8000× *g* for 10 min at 4 °C, and the supernatant was collected and kept on ice for subsequent analyses. Detailed sample preparation procedures are provided in the Supplementary Materials (Text S3).

Malondialdehyde (MDA, Cat. No. BC0020) and total protein (TP, Cat. No. PC0010) contents, as well as the activities of superoxide dismutase (SOD, Cat. No. BC0170), catalase (CAT, Cat. No. BC0200), peroxidase (POD, Cat. No. BC0090), reduced glutathione (GSH, Cat. No. BC1170), and glutathione S-transferase (GST, Cat. No. BC0350), were determined using commercial assay kits purchased from Beijing Solarbio Science & Technology Co., Ltd. (Beijing, China) strictly following the manufacturer’s instructions.

### 2.5. Determination of Intracellular ROS Levels

Intracellular reactive oxygen species (ROS) levels were determined using a DCFH-DA fluorescence assay. Approximately 0.1 g of fresh plant tissue was homogenized in 0.9 mL of pre-cooled PBS under ice-bath conditions, followed by centrifugation to obtain the supernatant. The DCFH-DA stock solution (10 mmol·L^−1^) was diluted 1000-fold with PBS to prepare the working solution. The sample was incubated with the probe at 37 °C in the dark. Fluorescence intensity was measured at excitation and emission wavelengths of 488 and 525 nm, respectively. ROS levels were expressed as relative fluorescence intensity (RFI) and normalized to soluble protein content (RFI·mg^−1^ protein).

### 2.6. Water Quality Parameters

Ammonium nitrogen (NH_4_^+^-N) was determined using the Nessler reagent spectrophotometric method following standard protocols (HJ 535-2009) [33], with absorbance measured at 420 nm. Total phosphorus (TP) was measured using the ammonium molybdate spectrophotometric method (GB/T 11893-1989) [34] at 700 nm using a UV-Vis spectrophotometer (Evolution 201, Thermo Fisher Scientific, Waltham, MA, USA).

### 2.7. Scanning Electron Microscopy Observation

Leaf samples were gently rinsed with deionized water to remove loosely attached impurities and then air-dried. The dried samples were mounted on aluminum stubs using conductive carbon tape and sputter-coated with gold using a Quorum Q150R ES sputter coater (Quorum Technologies Ltd., Lewes, UK) to improve conductivity. The surface morphology of epiphytic biofilms on the leaf samples was observed using a scanning electron microscope (SEM; ZEISS Sigma 500, Carl Zeiss AG, Oberkochen, Germany), following previously reported methods [35].

### 2.8. Epiphytic Biofilm Microbial Community and Transcriptomic Analysis

At the end of the experiment, approximately 2.0 g of fresh leaves were collected from each treatment and placed in 20 mL of 0.1 mol·L^−1^ phosphate-buffered saline (PBS, pH 7.4). The samples were subjected to ultrasonication for 10 min (160 W, 30 s intervals) to detach microorganisms from the leaf surface, followed by shaking incubation at 25 °C for 5 min. The resulting suspension was centrifuged at 10,000× *g* for 5 min at 4 °C using a refrigerated centrifuge (Heraeus Fresco 17, Thermo Fisher Scientific, Waltham, MA, USA). The cell pellets were collected and immediately stored at −80 °C. Microbial DNA extraction and sequencing were performed by Genedenovo Biotechnology Co., Ltd. (Guangzhou, China) using the Illumina MiSeq platform. Detailed information on sequencing procedures, quality control, and amplicon sequence variant (ASV) analysis is provided in the Supplementary Materials (Text S4). The composition and diversity of bacterial communities in the epiphytic biofilms were analyzed using the OmicShare interactive online platform (OmicsMaster, Guangzhou, China). Alpha diversity indices were calculated after rarefying all samples to the same sequencing depth. Transcriptomic analysis of plant samples was conducted as described in the Supplementary Materials (Text S5), where detailed information on RNA sequencing quality, data processing, and differential gene expression (DEGs) analysis are provided.

### 2.9. Statistical Analysis

All statistical analyses were performed using SPSS software (Version 25.0) and Origin (Version 2024). Data are presented as mean ± standard deviation (mean ± SD). Prior to analysis, data normality was assessed using the Shapiro–Wilk test, and homogeneity of variance was evaluated using Levene’s test. When the assumptions of parametric tests were met, one-way analysis of variance (ANOVA) was applied to compare differences among treatments, followed by Tukey’s post hoc test. For data that did not meet normality assumptions, the non-parametric Kruskal–Wallis test was used. Statistical significance was set at *p* < 0.05.

## 3. Results and Discussion

### 3.1. Effects of Microplastics on Plant Growth

**Figure 1:**
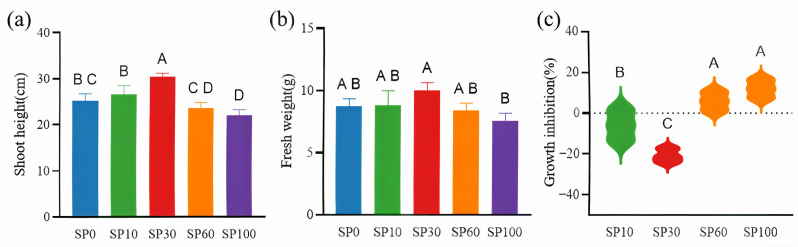
Growth responses of *Myriophyllum spicatum* under different polystyrene (PS) microplastic concentrations. (**a**) Shoot height; (**b**) Fresh weight; (**c**) Growth inhibition rate. Values represent mean ± SD. Different letters indicate significant differences among treatments (one-way ANOVA followed by Tukey’s test, *p* < 0.05). The dotted line in panel (**c**) indicates zero growth inhibition.

After 28 days of cultivation, shoot height and fresh weight of *Myriophyllum spicatum* were measured, and the growth inhibition rate was calculated to evaluate the effects of different treatments on plant growth. As shown in Figure 1a, compared with the control group, shoot height in the SP30 treatment significantly increased by 21.27% (*p* < 0.05), whereas it significantly decreased by 11.93% in the SP100 treatment (*p* < 0.05). The variation in fresh weight was generally consistent with that of shoot height (Figure 1b), although no significant differences were observed between treatments and the control (*p* > 0.05). The growth inhibition rate followed the order: SP30 < SP10 < SP60 < SP100 (Figure 1c), with SP30 showing the lowest and SP100 the highest inhibition. These results suggest a concentration-dependent response of *M. spicatum* to polystyrene microplastics under saline conditions. The stimulatory effect observed at low concentrations may be associated with adaptive physiological responses under mild stress, whereas the inhibitory effect at higher concentrations may be related to enhanced oxidative stress and disruption of cellular homeostasis. These interpretations are further discussed in the subsequent sections based on physiological and biochemical analyses.

### 3.2. Effects of Microplastics on Cell Membrane Integrity and Photosynthetic Pigments

**Figure 2:**
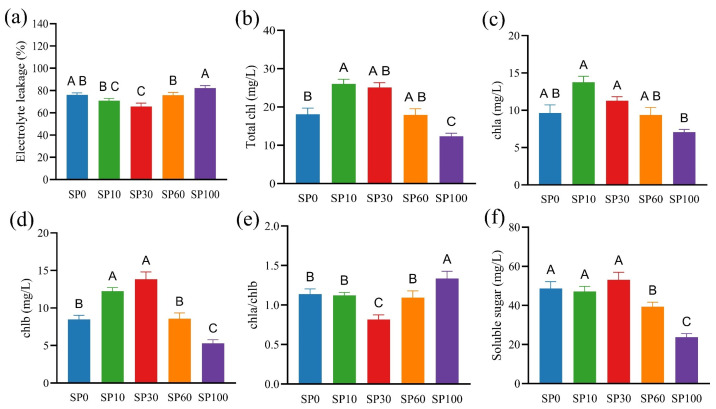
Physiological responses of *M. spicatum* under different PS microplastic concentrations. (**a**) Electrolyte leakage rate; (**b**) Total chlorophyll; (**c**) Chlorophyll a; (**d**) Chlorophyll b; (**e**) Chlorophyll a/b ratio; (**f**) Soluble sugars. Values are presented as mean ± SD. Different letters indicate significant differences among treatments (one-way ANOVA followed by Tukey’s test, *p* < 0.05).

Plant growth largely depends on photosynthetic efficiency, which is closely associated with cell membrane stability and photosynthetic pigment content. To evaluate the effects of microplastics on the photosynthetic system of *M. spicatum*, cell membrane integrity and photosynthesis-related parameters were measured under different treatments. As shown in Figure 2a, electrolyte leakage (EL) in the SP30 group was significantly lower than that in the control, whereas it was significantly higher in the SP100 group (*p* < 0.05). This pattern suggests that low concentrations of microplastics may be associated with reduced membrane damage, while higher concentrations are linked to increased membrane permeability.

Photosynthetic pigment contents exhibited an initial increase followed by a decrease. Total chlorophyll content was significantly higher in the SP10 group but significantly lower in the SP100 group compared with the control (*p* < 0.05; Figure 2b). Chlorophyll a content showed no significant differences between treatments and the control. However, it was significantly higher in the SP10 and SP30 groups than in the SP100 group (*p* < 0.05, Figure 2c). Chlorophyll b content was highest in the SP30 group and lowest in the SP100 group (Figure 2d). In addition, the chlorophyll a/b ratio in the SP100 group was significantly lower than that in the SP30 group (*p* < 0.05; Figure 2e). Soluble sugar content markedly decreased under high microplastic concentrations, with reductions of 19.22% and 51.21% in the SP60 and SP100 groups, respectively, compared with the control (*p* < 0.05; Figure 2f), while no significant differences were observed in the SP10 and SP30 groups (*p* > 0.05).

These results suggest that under low microplastic concentrations, *M. spicatum* can maintain a relatively stable membrane structure and higher levels of photosynthetic pigments, which are beneficial for light capture and carbon assimilation [36]. In contrast, high concentrations of microplastics disrupt membrane stability and interfere with the photosynthetic system, potentially reducing light absorption and energy transfer efficiency, thereby affecting the accumulation of photosynthetic products. This may explain the decreased soluble sugar content and growth inhibition observed in the SP100 treatment.

Microscopic observations (Figure S5) further supported these findings, showing that leaf tissues in the SP30 group maintained relatively intact cellular morphology, whereas those in the SP100 group exhibited visible structural disruption, including cell deformation and chloroplast disorganization, consistent with the increased membrane damage and reduced photosynthetic performance under high microplastic concentrations.

### 3.3. Oxidative Stress Induced by Membrane and Photosynthetic Damage

Damage to cell membranes and impairment of the photosynthetic system are typically associated with the accumulation of ROS, which are required to be scavenged by the plant antioxidant system. To evaluate the extent of oxidative damage and antioxidant defense responses, oxidative and antioxidant-related parameters in *M. spicatum* were measured under different treatments.

**Figure 3:**
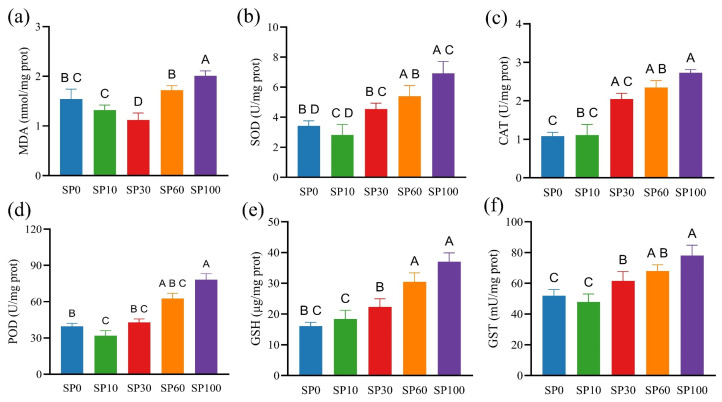
Antioxidant responses of *M. spicatum* under different PS microplastic concentrations. (**a**) MDA; (**b**) SOD; (**c**) CAT; (**d**) POD; (**e**) GSH; (**f**) GST. Values are presented as mean ± SD. Different letters indicate significant differences among treatments (one-way ANOVA followed by Tukey’s test, *p* < 0.05).

As shown in Figure S6, ROS levels increased progressively with increasing microplastic concentrations, with significantly higher values observed in the SP60 and SP100 groups compared to the control (*p* < 0.05). Notably, ROS levels were also elevated in the SP30 group. However, as shown in Figure 3a, MDA content significantly decreased in the SP30 group but increased markedly in the SP100 group (*p* < 0.05), indicating that moderate ROS accumulation may not necessarily lead to oxidative damage. The activities of SOD, CAT, and POD generally increased with rising microplastic concentrations, with significantly higher values observed in the SP100 group compared to the control (*p* < 0.05; Figure 3b–d). In addition, GSH content and GST activity also showed increasing trends. Compared with the control, GSH content was significantly elevated in the SP60 and SP100 groups, while GST activity was significantly increased in the SP30, SP60, and SP100 groups (*p* < 0.05; Figure 3e,f).

Under high microplastic concentrations, the simultaneous increase in ROS and MDA levels, together with enhanced antioxidant responses, indicates that the plants were subjected to pronounced oxidative stress. In contrast, the elevated ROS levels observed in the SP30 group, accompanied by reduced MDA content and increased antioxidant activity, suggest that ROS at moderate levels may function as a signaling factor rather than causing oxidative damage. When cell membrane integrity or photosynthetic processes are disrupted by environmental stress, the electron transport chain can become imbalanced, leading to ROS accumulation and subsequent activation of antioxidant defense systems [37]. In addition, salinity stress can elevate oxidative stress in plants by affecting membrane stability and energy metabolism [38]. In this study, high concentrations of microplastics may have further intensified this oxidative stress, thereby stimulating increased antioxidant enzyme activities and the glutathione-related antioxidant system to facilitate ROS scavenging.

### 3.4. Changes in Nitrogen and Phosphorus Uptake Under Cumulative Physiological Stress

**Figure 4:**
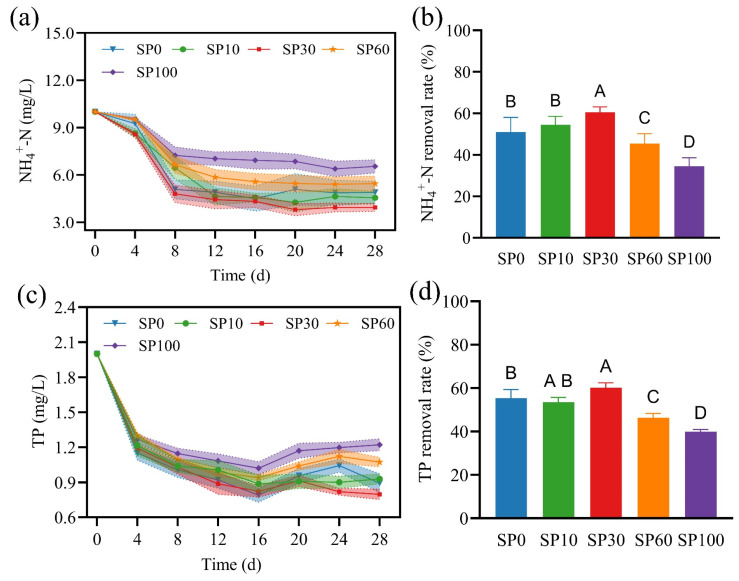
Changes in nutrient concentrations during the experimental period. (**a**) NH_4_^+^-N concentration; (**b**) NH_4_^+^-N removal rate; (**c**) TP concentration; (**d**) TP removal rate. Values are presented as mean ± SD. Different letters indicate significant differences among treatments (one-way ANOVA followed by Tukey’s test, *p* < 0.05).

We monitored the dynamic changes in ammonium nitrogen (NH_4_^+^-N) and total phosphorus (TP) concentrations in the water under different microplastic treatments to evaluate the nutrient removal capacity of *M. spicatum*. The results showed that microplastic exposure significantly affected the removal of NH_4_^+^-N and TP. During the initial stage (0–8 d), NH_4_^+^-N concentrations decreased in all groups, with differences among treatments becoming apparent after day 4 (Figure 4a). In the middle stage (8–20 d), the decline in NH_4_^+^-N slowed, with the SP30 group consistently showing lower concentrations than the control, whereas the SP60 and SP100 groups maintained higher levels. By day 28, NH_4_^+^-N removal efficiency followed the order: SP30 (60.52%) > SP10 (55.13%) > SP0 (51.68%) > SP60 (47.21%) > SP100 (35.87%) (Figure 4b), with SP30 showing the highest and SP100 the lowest removal efficiency. TP showed a similar trend to NH_4_^+^-N, decreasing rapidly during 0–16 d and then stabilizing (Figure 4c). The SP30 treatment exhibited significantly lower TP concentrations than the control, whereas SP60 and SP100 maintained relatively higher levels. By day 28, TP removal efficiency ranked as follows: SP30 (61.20%) > SP0 (59.60%) > SP10 (54.70%) > SP60 (46.30%) > SP100 (38.10%) (Figure 4d).

Nitrogen and phosphorus uptake in plants requires energy and depends on membrane transport processes [39]. In this study, high concentrations of microplastics were accompanied by pronounced oxidative stress, which may lead to increased energy allocation to antioxidant defense, thereby potentially reducing the energy available for growth and nutrient uptake. In addition, stress-induced alterations in membrane structure may impair transmembrane transport processes, which could contribute to reduced nutrient uptake efficiency. In contrast, under low microplastic concentrations, plants exhibited relatively minor physiological disturbances and maintained higher nutrient removal efficiency.

### 3.5. Effects of Microplastic Stress on the Transcriptome

#### 3.5.1. Transcriptome Overview

Based on the physiological results described above, the SP30 group exhibited better growth performance and higher nitrogen and phosphorus removal efficiency, whereas the SP100 group showed pronounced growth inhibition and oxidative stress responses. To elucidate the molecular regulatory mechanisms underlying these physiological responses, transcriptomic analysis was conducted on leaves of *M. spicatum* from the control group (SP0, 0 mg·L^−1^), the low-concentration microplastic treatment (SPL, 30 mg·L^−1^), and the high-concentration treatment (SPH, 100 mg·L^−1^).

**Figure 5:**
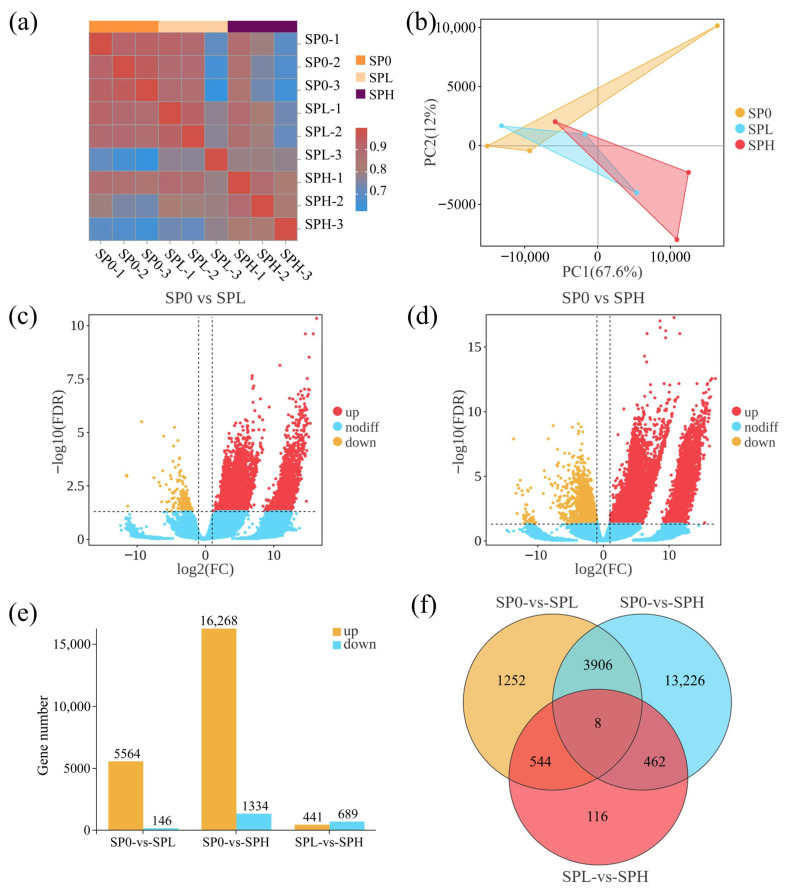
Transcriptomic analysis of *M. spicatum* leaves under different treatments. (**a**) Sample correlation heatmap; (**b**) Principal component analysis (PCA); (**c**,**d**) Volcano plots of differentially expressed genes (DEGs); (**e**) Number of up- and down-regulated DEGs; (**f**) Venn diagram of DEGs among treatments. The vertical dotted lines in panels (**c**,**d**) indicate the log_2_ fold-change thresholds, and the horizontal dotted line indicates the statistical significance threshold (FDR = 0.05).

Sequencing quality assessment showed that the correlation coefficients among biological replicates were all higher than 0.76 (Figure 5a). The Q20 and Q30 values ranged from 99.09% to 99.30% and 96.77% to 97.56%, respectively (Table S4), both exceeding the quality control thresholds (>85% and >80%). These results indicate that the sequencing data were of high quality and suitable for subsequent analyses. Principal component analysis (PCA) clearly separated the SP0, SPL, and SPH groups (Figure 5b), indicating that gene expression patterns were significantly altered under different microplastic treatments.

Differential expression analysis (|log_2_fold change| ≥ 2 and FDR < 0.05) showed that, compared with SP0, a large number of differentially expressed genes (DEGs) were identified in the SPH group, including 16,268 up-regulated and 1334 down-regulated genes. In contrast, the SPL group exhibited substantially fewer DEGs, with 5564 up-regulated and 146 down-regulated genes (Figure 5c–e). Venn diagram analysis revealed that 17,602 DEGs were shared between SPH and SP0, whereas only 5710 DEGs were shared between SPL and SP0 (Figure 5f). In addition, the numbers of unique DEGs for SP0 vs. SPH, SP0 vs. SPL, and SPL vs. SPH were 13,226, 1252, and 116, respectively. The markedly higher number of DEGs in the SP0 vs. SPH comparison suggests that high-concentration microplastic exposure induced more substantial transcriptomic alterations.

#### 3.5.2. Differentially Expressed Genes

Among the differentially expressed genes (DEGs), multiple genes associated with photosynthesis, oxidative stress, energy metabolism, lipid metabolism, and nitrogen and phosphorus metabolism exhibited significant changes (Table S5).

**Figure 6:**
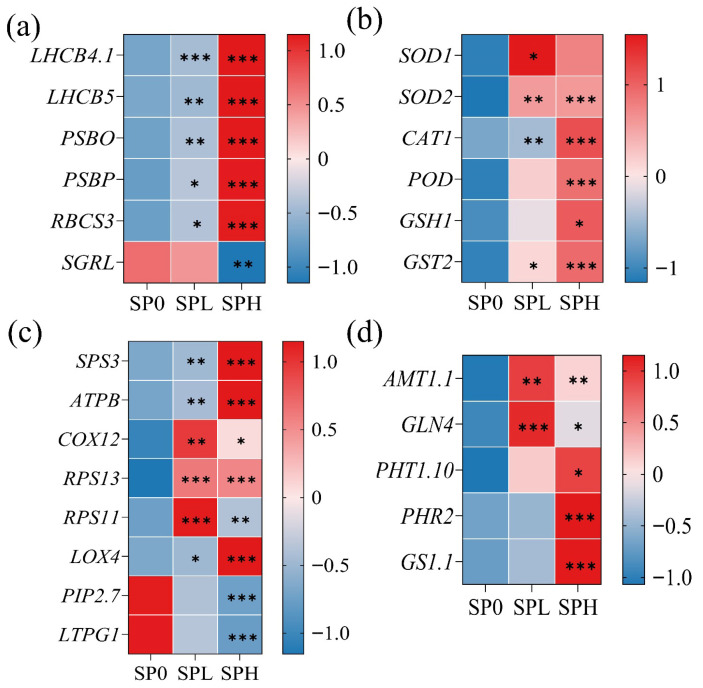
Heatmap of representative DEGs in *M. spicatum* leaves under different treatments. (**a**) Photosynthesis-related genes; (**b**) Oxidative stress-related genes; (**c**) Energy metabolism-related genes; (**d**) Nitrogen and phosphorus metabolism-related genes. Colors represent relative gene expression levels (z-score). Asterisks indicate statistical significance based on FDR values (* FDR < 0.05, ** FDR < 0.01, *** FDR < 0.001).

For photosynthesis-related genes (Figure 6a), compared with the control, the SPH group showed significant up-regulation of light-harvesting complex genes (*LHCB4.1* and *LHCB5*), oxygen-evolving complex genes (*PSBO* and *PSBP*), and the key carbon fixation gene *RBCS3*. In contrast, these genes showed no significant differences between the SPL and control groups, indicating no significant transcriptional changes under low microplastic exposure. In addition, the chlorophyll degradation-related gene *SGRL* was significantly down-regulated in the SPH group. These changes in gene expression are more likely to reflect a compensatory response aimed at maintaining or repairing photosynthetic function under stress, rather than a direct enhancement of photosynthetic capacity [40].

For antioxidant-related genes (Figure 6b), *SOD1* was significantly up-regulated in the SPL group but only slightly increased in the SPH group compared with the control, suggesting that low microplastic concentrations may preferentially activate cytosolic antioxidant systems [41]. *SOD2* was significantly up-regulated in both SPL and SPH groups, indicating enhanced mitochondrial ROS scavenging capacity. Moreover, in the SPH group, catalase gene *CAT1*, peroxidase gene *POD*, glutathione synthesis gene *GSH1*, and glutathione S-transferase gene *GST2* were all significantly up-regulated. This is consistent with the previous result of increased antioxidant enzyme activity, suggesting that plants may enhance their ability to remove and detoxify ROS by up-regulating antioxidant enzymes and genes related to the glutathione cycle, thereby alleviating oxidative damage.

For energy metabolism and lipid-related genes (Figure 6c), the SPL group showed significant up-regulation of respiratory chain gene *COX12* and protein synthesis-related genes *RPS13* and *RPS11*, suggesting enhanced mitochondrial respiration and ATP production, which may support antioxidant defense and protein synthesis [42]. In the SPH group, sucrose synthesis gene *SPS3* and ATP synthase subunit gene *ATPB* were significantly up-regulated. However, soluble sugar content was lowest under this treatment, indicating that although sucrose synthesis and energy metabolism pathways were transcriptionally enhanced, sugars did not accumulate effectively. This may be because more carbon assimilates were consumed for antioxidant defense, ion transport, and membrane repair processes [43]. In addition, the lipid peroxidation-related gene *LOX4* was significantly up-regulated, whereas the aquaporin gene *PIP2.7* and the lipid transport protein gene *LTPG1* were significantly down-regulated in the SPH group. Aquaporins play a key role in regulating membrane water permeability and maintaining cellular water balance [44]. Therefore, the down-regulation of *PIP2.7* may indicate reduced water transport capacity and altered osmotic regulation under high microplastic exposure. Meanwhile, the decreased expression of *LTPG1*, together with the up-regulation of *LOX4*, may reflect enhanced lipid peroxidation and disturbances in membrane lipid transport. These changes may contribute to disrupted cellular homeostasis, which could further limit carbon assimilation and result in reduced soluble sugar accumulation.

For nitrogen and phosphorus metabolism (Figure 6d), ammonium transporter gene *AMT1.1* and glutamine synthetase gene *GLN4* were significantly up-regulated in the SPL group, suggesting that plants may enhance ammonium uptake and assimilation under low microplastic exposure [45]. In contrast, *GS1.1*, which is involved in intracellular ammonium recycling, was significantly up-regulated in the SPH group, indicating that plants may maintain nitrogen balance by strengthening internal nitrogen cycling under stress conditions [46]. In addition, phosphate transporter gene *PHT1.10* and phosphate regulatory factor *PHR2* were significantly up-regulated in the SPH group. Under conditions of limited phosphorus availability or reduced uptake capacity, plants often activate phosphate starvation responses, suggesting that plants may enhance phosphorus acquisition through increased transport and regulatory capacity [47].

Overall, the transcriptomic results provide molecular-level evidence supporting the observed physiological changes, indicating that microplastic stress may influence energy metabolism and nutrient uptake in *M. spicatum* by regulating genes involved in photosynthesis, antioxidant defense, and nitrogen and phosphorus metabolism.

#### 3.5.3. GO and KEGG Enrichment Analysis

To clarify the biological processes associated with these DEGs and their functional differences under different treatments, GO and KEGG enrichment analyses were performed for the SPL and SPH groups.

**Figure 7:**
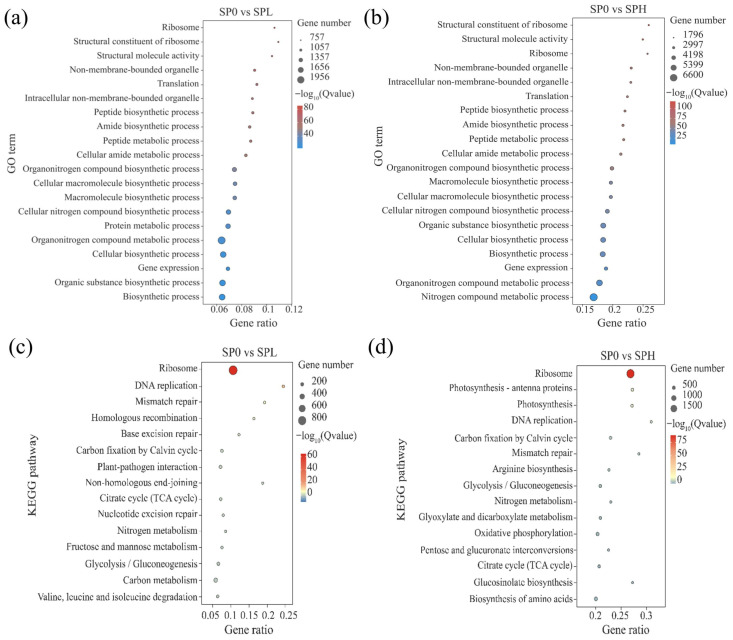
Functional enrichment analysis of differentially expressed genes in *M. spicatum* leaves. (**a**,**b**) GO enrichment analysis; (**c**,**d**) KEGG pathway enrichment analysis.

The top 20 enriched GO terms for each group are shown in Figure 7a,b. DEGs in both SPL and SPH groups were mainly enriched in protein synthesis-related categories, including ribosome, translation, and peptide biosynthetic process. These processes are commonly associated with enhanced protein turnover and stress adaptation under adverse conditions. In addition, the SPL group was also enriched in protein metabolic process, whereas the SPH group showed further enrichment in nitrogen compound metabolic process. The enrichment of nitrogen-related processes in the SPH group may reflect increased demand for amino acid biosynthesis and metabolic adjustment under stronger stress conditions. Moreover, the overall gene ratio was higher in the SPH group than in the SPL group, indicating a more extensive transcriptional response. Collectively, these results suggest that low microplastic exposure mainly triggers adjustments in basic protein metabolism, whereas high microplastic exposure induces more intensive metabolic reprogramming to cope with stress.

The top 15 enriched KEGG pathways for each group are presented in Figure 7c,d. KEGG analysis showed that DEGs in both groups were involved in pathways such as ribosome, DNA replication, carbon fixation by the Calvin cycle, and nitrogen metabolism, although the enrichment patterns differed between treatments. The SPL group was mainly enriched in DNA repair-related pathways, including mismatch repair, homologous recombination, and base excision repair, as well as in the citrate cycle (TCA cycle) and carbon metabolism pathways. These results indicate that under low microplastic exposure, *M. spicatum* may maintain cellular stability by sustaining basic carbon metabolism and repairing molecular damage [48]. In contrast, the SPH group was significantly enriched in pathways such as photosynthesis-antenna proteins, photosynthesis, glycolysis/gluconeogenesis, and oxidative phosphorylation. This suggests that under high microplastic stress, photosynthesis-related processes are affected, and pathways associated with energy metabolism and nitrogen metabolism may be involved in the plant stress response.

### 3.6. Response of the Phyllosphere Microbial Community

#### 3.6.1. Biofilm Morphology

**Figure 8:**
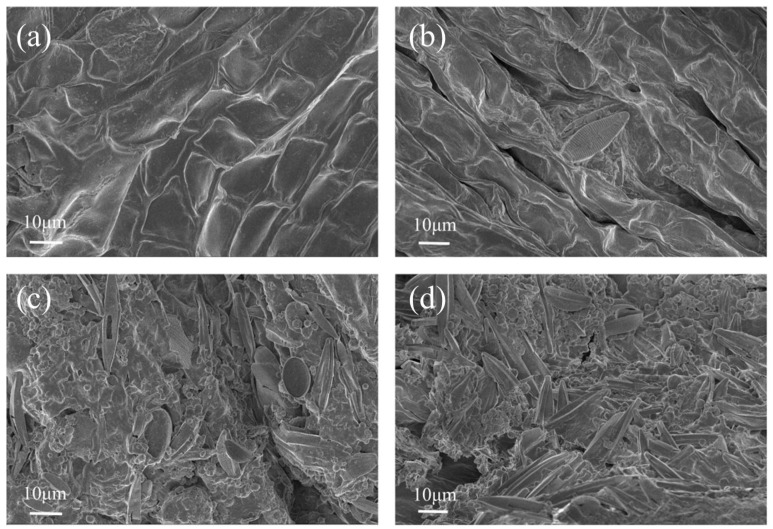
SEM images of biofilms on the leaves of *M. spicatum* under different PS microplastic treatments. (**a**) SP0; (**b**) SP10; (**c**) SP30; (**d**) SP100.

To investigate the effects of microplastic stress on the phyllosphere microbial community of *M. spicatum*, scanning electron microscopy (SEM) was used to observe the structure of epiphytic biofilms on leaf surfaces under different treatments. The results showed that the leaf surface in the control group was relatively smooth, whereas abundant spherical and rod-shaped microorganisms were observed aggregating on the leaf surfaces in the SP30 and SP100 treatments, forming more complex biofilm structures (Figure 8). Previous studies have suggested that microplastic particles, due to their large specific surface area and rough surface characteristics, can provide substrates for microbial attachment, thereby promoting microbial colonization and aggregation [49]. Accordingly, under the saline conditions of this study, microplastic exposure may have promoted the formation of epiphytic biofilms on leaf surfaces.

#### 3.6.2. Community Structure and Diversity

**Figure 9:**
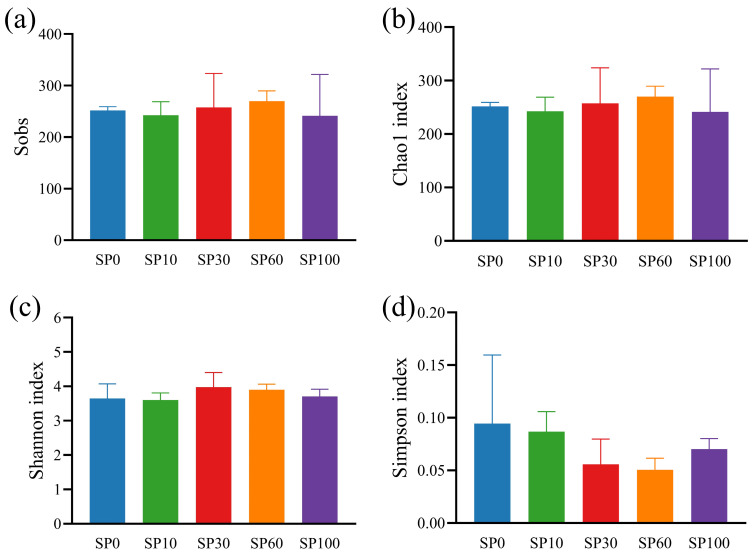
Alpha diversity indices of phyllosphere microbial communities associated with *M. spicatum*. (**a**) Sobs index; (**b**) Chao1 index; (**c**) Shannon index; (**d**) Simpson index. Values are presented as mean ± SD. No significant differences were detected among treatments (Kruskal–Wallis test, *p* > 0.05).

The phyllosphere microbial community was analyzed using 16S rRNA high-throughput sequencing, and sequencing quality metrics are summarized in Table S6. A total of 44,146–62,457 effective tags were obtained, with consistent sequence lengths across all samples, indicating that the data quality was sufficient for subsequent analyses. After denoising, 171–333 amplicon sequence variants (ASVs) were identified, with comparable ASV numbers across treatments. Alpha diversity analysis (Figure 9) showed that Sobs and Chao1 indices ranged from 171 to 333, with no significant differences among treatments (Kruskal–Wallis test, *p* > 0.05), suggesting that microplastic exposure had limited effects on species richness in the phyllosphere microbial community. Similarly, Shannon and Simpson indices did not differ significantly among treatments (Kruskal–Wallis test, *p* > 0.05), indicating that overall community diversity and evenness remained stable.

**Figure 10:**
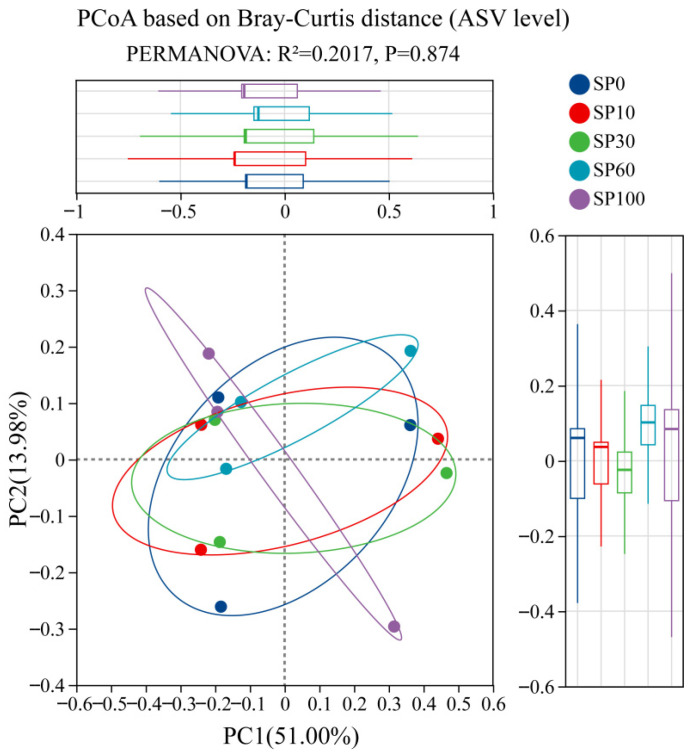
Principal coordinate analysis (PCoA) based on Bray–Curtis distances at the ASV level showing the differences in phyllosphere microbial community structure associated with *M. spicatum* under different PS treatments. The dotted lines indicate the zero positions of PC1 and PC2.

To further assess differences in community structure, principal coordinates analysis (PCoA) based on Bray–Curtis distances at the ASV level was performed (Figure 10). The results showed substantial overlap among samples from different treatments, with no clear separation observed. Consistently, Adonis (PERMANOVA) analysis indicated that microplastic exposure did not significantly affect community structure (R^2^ = 0.2017, *p* = 0.874). Boxplots of within-group dispersion also showed similar levels of community variability across treatments. Overall, these results suggest that, under saline conditions, different concentrations of microplastics did not significantly alter the overall structure of the phyllosphere microbial community associated with *M. spicatum*.

#### 3.6.3. Community Composition

**Figure 11:**
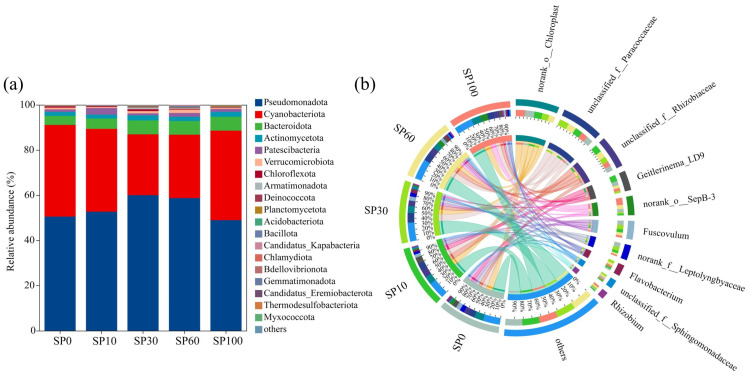
Relative abundance of phyllosphere microbial communities associated with *M. spicatum*. (**a**) Phylum-level composition; (**b**) Genus-level composition.

At the phylum level (Figure 11a), the epiphytic microbial community on *M. spicatum* leaves was mainly composed of Pseudomonadota and Cyanobacteriota. With increasing microplastic concentrations, the relative abundance of Pseudomonadota showed an initial increase followed by a decrease, rising from 50.49% in the control group to 59.99% in the SP30 group and then declining to 48.92% in the SP100 group. In contrast, Cyanobacteriota exhibited a decreasing trend followed by recovery, reaching a minimum of 26.93% in the SP30 group and increasing to 39.65% in the SP100 group. In addition, the relative abundance of Bacteroidota generally increased with increasing microplastic concentrations. Other phyla, including Patescibacteria, Verrucomicrobiota, and Chloroflexota, were present at relatively low abundances and contributed minimally to overall community composition. Overall, the dominant phyla remained relatively stable across treatments, consistent with the diversity analysis results. This suggests that microplastic exposure mainly influenced the relative abundances of specific taxa rather than causing substantial shifts in overall community structure.

At the genus level (Figure 11b), the dominant taxa across treatments were unclassified_f__Paracoccaceae, unclassified_f__Rhizobiaceae, and *Geitlerinema*_LD9. Among them, unclassified_f__Paracoccaceae showed the highest relative abundance in the SP30 group, whereas unclassified_f__Rhizobiaceae and *Geitlerinema*_LD9 were most abundant in the SP10 group. Previous studies have shown that members of Paracoccaceae and Rhizobiaceae are commonly found in plant-associated microbial communities and may be associated with nitrogen cycling and host stress responses [50,51]. In addition to dominant genera, several moderately abundant taxa also exhibited notable changes across treatments. For instance, norank_o__SepB-3 and *Rhizobium* increased in relative abundance under high microplastic exposure, whereas an unclassified taxon annotated as *Fuscovulum* showed a decreasing trend with increasing microplastic concentrations. Phyllosphere microbial communities are known to shift in response to changes in plant physiological status and leaf microenvironment [52,53]. Therefore, these changes in both classified and unclassified taxa may be associated with plant physiological status or nutrient-related processes in the phyllosphere. Based on these observations, further correlation analysis was conducted to explore the relationships between microbial taxa and plant physiological parameters.

#### 3.6.4. Correlations Between Plant Physiological Traits and Key Microbial Genera

To explore potential associations between microbial genera and plant physiological functions, Spearman correlation analysis was performed based on genus-level abundance data, linking dominant taxa with plant physiological parameters and nutrient removal efficiency (Tables S7 and S8). The results showed that norank_o__SepB-3 and *Rhizobium* were significantly negatively correlated with total chlorophyll content and positively correlated with multiple antioxidant enzyme activities, indicating statistical associations between these taxa and plant physiological parameters, which may reflect links with plant stress-related responses. In contrast, *Fuscovulum* showed significant positive correlations with NH_4_^+^-N and TP removal efficiencies, but negative correlations with the chlorophyll a/b ratio and MDA content. No significant correlations were observed between other microbial genera and plant physiological indicators.

**Figure 12:**
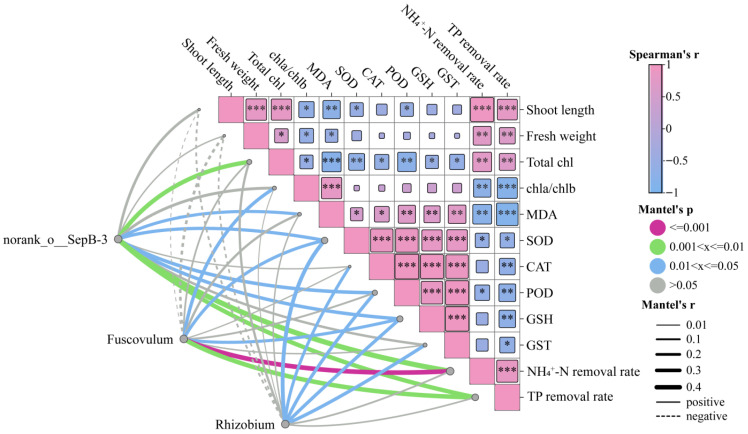
Correlation analysis between plant physiological indices and key microbial genera. Spearman correlation analysis was used to evaluate relationships between plant physiological parameters and microbial genera, and Mantel tests were performed to assess overall associations between key microbial taxa and plant physiological traits. Asterisks indicate statistical significance levels (* *p* < 0.05, ** *p* < 0.01, *** *p* < 0.001).

To further evaluate the overall associations between key microbial taxa and host physiological functions, Mantel tests were conducted for norank_o__SepB-3, *Rhizobium*, and *Fuscovulum*, which showed significant correlations in the Spearman analysis (Figure 12, Table S9). norank_o__SepB-3 was significantly positively correlated with total chlorophyll content, antioxidant enzyme activities (including SOD, POD, and GSH), as well as NH_4_^+^-N and TP removal efficiencies, suggesting that this taxon may be associated with both stress responses and nutrient removal processes. *Fuscovulum* was mainly positively correlated with NH_4_^+^-N and TP removal, whereas *Rhizobium* showed significant positive correlations with antioxidant enzyme activities but no significant correlations with plant growth or nutrient removal efficiency (*p* > 0.05).

By integrating plant physiological results with microbial abundance patterns, it can be inferred that different microbial taxa exhibited distinct response patterns under microplastic stress. With increasing microplastic concentrations, oxidative stress levels in plants increased, accompanied by higher relative abundances of norank_o__SepB-3 and *Rhizobium*, which were positively correlated with antioxidant enzyme activities. This suggests that these taxa may be linked to plant stress responses. Previous studies have shown that plant-associated microbial communities can influence plant adaptation to environmental stress by modulating host redox status or participating in antioxidant processes [54,55]. Among them, *Rhizobium* is widely distributed in plant-associated microbial communities and has been reported to contribute to plant stress tolerance [56]. In contrast, the relative abundance of *Fuscovulum* decreased with increasing microplastic concentrations and was positively correlated with nutrient removal efficiency, suggesting that this taxon may be associated with nutrient transformation processes and plant health status. However, its taxonomic identity remains to be further resolved. Previous studies have indicated that some unclassified bacterial taxa may participate in organic matter degradation and nitrogen cycling, thereby influencing nutrient dynamics in aquatic systems [57]. In this study, microplastic exposure reduced photosynthetic performance and nutrient uptake capacity in plants, which may alter the availability of organic substrates and nutrient conditions in the phyllosphere. Such changes could be unfavorable for taxa associated with nutrient cycling and may be associated with changes in phyllosphere microbial composition, which may have implications for nutrient dynamics.

Overall, although microplastic exposure did not significantly alter the overall structure of the phyllosphere microbial community, shifts in genus-level relative abundances were observed under different treatments. By integrating physiological responses, transcriptomic profiles, and phyllosphere microbial data, a multi-level response framework of *M. spicatum* under microplastic stress can be proposed. At the molecular level, microplastic exposure induced differential expression of genes related to photosynthesis, antioxidant defense, and nitrogen and phosphorus metabolism. The up-regulation of antioxidant-related genes (*SOD1*, *SOD2*, *CAT1*, *GSH1*, and *GST2*) was consistent with the observed increase in antioxidant enzyme activities. Meanwhile, changes in photosynthesis-related genes, together with the decline in chlorophyll content and soluble sugars, suggest that photosynthetic processes and carbon assimilation may be affected. Given that *M. spicatum* is a submerged macrophyte with limited stomatal regulation, the observed decline in photosynthetic performance is more likely associated with alterations in photosynthetic electron transport and carbon fixation processes. At the physiological level, these molecular changes were accompanied by enhanced lipid peroxidation, activation of antioxidant systems, reduced photosynthetic pigment levels, and decreased nutrient removal efficiency. This pattern may reflect a shift in plant metabolic strategy from growth-oriented processes toward stress defense and maintenance of cellular homeostasis. Under such conditions, carbon assimilates and energy may be preferentially allocated to antioxidant regulation, membrane repair, and related metabolic processes, potentially limiting their availability for growth and nutrient uptake. In addition, reactive oxygen species may play a dual role, acting as signaling molecules under moderate stress but contributing to oxidative damage under higher microplastic exposure, as indicated by increased MDA levels and antioxidant responses.

Furthermore, these physiological and molecular changes may alter the phyllosphere microenvironment, thereby influencing microbial responses. Although no significant changes in community structure were observed, shifts at the genus level were detected. Specifically, norank_o__SepB-3 and *Rhizobium* increased under high microplastic exposure and showed positive correlations with antioxidant enzyme activities, indicating statistical associations with plant physiological status. In contrast, *Fuscovulum* showed a decreasing trend with increasing microplastic concentration and was positively correlated with nutrient removal efficiency, suggesting potential links with nutrient-related processes. These findings collectively suggest that microplastic stress may influence plant–microbe interactions indirectly through coordinated physiological and molecular responses, rather than through large-scale changes in microbial community structure. It should be noted that this study has several limitations. The analysis was primarily based on physiological indicators, transcriptomic data, and microbial community composition, while direct measurements of photosynthetic processes and carbon allocation were not conducted. In addition, microbial functional roles were inferred from correlation analysis and literature, rather than experimentally validated. Furthermore, dissolved oxygen was not measured, and its potential influence on plant physiology and microbial processes remains to be clarified. Future studies integrating multi-omics analyses and functional validation approaches are needed to further elucidate these mechanisms.

## 4. Conclusions

This study elucidates the effects of polystyrene (PS) microplastics on the submerged macrophyte *Myriophyllum spicatum* under saline conditions. The results demonstrate a clear concentration-dependent pattern, characterized by low-dose stimulation and high-dose inhibition. Low concentrations of PS maintained membrane stability and photosynthetic performance, and enhanced nutrient assimilation and antioxidant capacity, thereby supporting plant growth. In contrast, high concentrations caused membrane damage, photosynthetic inhibition, oxidative stress, and reduced nitrogen and phosphorus uptake, indicating evident phytotoxic effects. Microplastic exposure promoted epiphytic biofilm formation on leaf surfaces without significantly altering overall microbial community structure, but shifted specific functional taxa associated with antioxidant responses and nutrient removal. Overall, under saline conditions, PS microplastics exert phytotoxic effects on submerged plants by disrupting membrane integrity, photosynthetic processes, and redox homeostasis, while being associated with changes in functionally relevant microbial groups. These findings highlight the importance of considering combined environmental stressors and plant–microbe interactions in ecological risk assessment of microplastics.

## Data Availability

The data presented in this study can be accessed at https://github.com/datashare768/Microplastics (accessed on 7 May 2026).

## Supplementary Materials

The following supporting information can be downloaded at https://www.mdpi.com/article/10.3390/toxics14050416/s1. Text S1: Experiment solution osmotic potential calculation method; Text S2: Electrolyte Leakage; Text S3: Total protein and enzyme activities; Text S4: Experimental procedure for microbiome diversity amplicon sequencing; Text S5: Transcriptome analysis; Figure S1: FTIR spectrum of polystyrene microplastics; Figure S2: Morphology and size distribution of polystyrene microplastics; Figure S3: Zeta potential distribution of polystyrene microplastics in deionized water; Figure S4: Growth status of *Myriophyllum spicatum* at day 14 of the experiment; Figure S5: Optical microscopy images of leaf tissues of M. spicatum under different microplastic treatments; Figure S6: ROS levels under different treatments; Table S1: The components of sea salt; Table S2: The components of Hoagland medium; Table S3: Nutrient concentrations of the Pearl River in China; Table S4: Summary of the transcriptome sequencing data; Table S5: Expression levels and statistical significance of DEGs; Table S6: Summary statistics of sequencing data for all samples; Table S7: Spearman correlation coefficients (r value) between physiological indices and key microbial genus; Table S8: Spearman correlation coefficients (*p* value) between physiological indices and key microbial genus; Table S9: Mantel test results showing correlations between key microbial genera and plant physiological traits.
