# Supplementary material for: Phytotoxic Mechanisms of Polystyrene Microplastics in Myriophyllum spicatum Under Saline Conditions: Insights from Physiology, Transcriptomics, and Phyllosphere Microbiota
Source: Toxics. 2026 May 10;14(5):416. doi: 10.3390/toxics14050416 (PMC13211750; doi:10.3390/toxics14050416)
Supplement: Supplementary file 1 [file toxics-14-00416-s001.zip › toxics-4245020-supplementary.pdf]

Supplementary Information

Phytotoxic Mechanisms of Polystyrene Microplastics in *Myriophyllum spicatum* Under Saline Conditions: Insights from Physiology, Transcriptomics, and Phyllosphere Microbiota

Junyu Xuan [1], Jinquan Wan [1,*], Lanhui Wen [1], Yan Wang [1] and Ji Shiming [2]

[1] Department of Environment and Energy, South China University of Technology, Guangzhou 510006, China
[2] Guangdong Shunkong Zihua Technology Co., Ltd., Foshan 528300, China
* Correspondence: ppjqwan@scut.edu.cn

Text List

Text S1. Experiment solution osmotic potential calculation method.

Text S2. Electrolyte Leakage.

Text S3. Total protein and enzyme activities.

Text S4. Experimental procedure for microbiome diversity amplicon sequencing.

Text S5. Transcriptome analysis.

Figure List

Figure S1. FTIR spectrum of polystyrene microplastics.

Figure S2. Morphology and size distribution of polystyrene microplastics.

Figure S3. Zeta potential distribution of polystyrene microplastics in deionized water.

Figure S4. Growth status of *Myriophyllum spicatum* at day 14 of the experiment.

Figure S5. Optical microscopy images of leaf tissues of *M. spicatum* under different microplastic treatments.

Figure S6. ROS levels under different treatments.

Table List

Table S1. The components of sea salt.

Table S2. The components of Hoagland medium.

Table S3. Nutrient concentrations of the Pearl River in China.

Table S4. Summary of the transcriptome sequencing data.

Table S5. Expression levels and statistical significance of DEGs.

Table S6. Summary statistics of sequencing data for all samples.

Table S7. Spearman correlation coefficients (r value) between physiological indices and key microbial genus.

Table S8. Spearman correlation coefficients (p value) between physiological indices and key microbial genus.

Table S9. Mantel test results showing correlations between key microbial genera and plant physiological traits.

Text S1. Experiment solution osmotic potential calculation method

The osmotic potential of the experimental solution (Ψ_s) is calculated using the following equation for dilute solutions:

$$\Psi_{s} = -CRT$$

Where C is the molar concentration of solute (mol·L^{-1}), R is the gas constant (8.314×10^{-3} MPa·L·mol^{-1}·K^{-1}), and T is the temperature (298.15 K, corresponding to 25°C).

In this study, the experimental solution includes artificial sea salt (formulation shown in Table S1), along with added NH_4Cl and KH_2PO_4. The process for calculating the total ion molar concentration is as follows:

Contribution of Artificial Seawater: Based on the mass proportion of each component in Table S1, the molar concentration of each salt in the 0.5% (w/v, 5 g·L^{-1}) artificial seawater solution was calculated. The ion dissociation number was then multiplied to obtain the total ion molar concentration contribution of the seawater, which was 0.132 mol·L^{-1}.

Contribution of Nutrient Salts: The added NH_4Cl (which results in a NH_4^+-N concentration of 10 mg·L^{-1}) and KH_2PO_4 (which results in a TP concentration of 2 mg·L^{-1}) contributed total ion molar concentrations of 0.0014 mol·L^{-1} and 0.00013 mol·L^{-1}, respectively.

Total Ion Molar Concentration: By summing the ion concentrations of all the solutes mentioned above, the total ion molar concentration of the experimental solution was approximately 0.134 mol·L^{-1}.

Substituting into the formula, the osmotic potential of the solution at 25°C was calculated to be approximately -0.33 MPa. This osmotic potential simulates estuarine low-salinity environments and does not cause significant osmotic stress to *Myriophyllum spicatum*, ensuring normal plant growth.

Text S2. Electrolyte Leakage

Cell membrane integrity was assessed by measuring leaf electrolyte leakage (EL). Leaves from each treatment were sampled, and 10 mm leaf discs were punched (10 replicates per treatment) and placed into test tubes containing 10 mL of distilled water. The samples were incubated at room temperature with shaking at 250 rpm for 4 h. After incubation, the initial electrical conductivity (EC1) was measured using a conductivity meter (P902, Shanghai Youke Instrument Co., Ltd.). The samples were then autoclaved at 121°C for 15 min, cooled to room temperature, and the final electrical conductivity (EC2) was recorded. Electrolyte leakage (EL) was calculated using the following formula; a higher EL value indicates more severe damage to cell membrane integrity.

$$\mathrm{EC}\ (\%)=\left(\frac{\mathrm{EC1}}{\mathrm{EC2}}\right)\times 100 \qquad \text{(S1)}$$

Text S3. Total protein and enzyme activities

On the 28th day of the experiment, 1.0 g of leaf tissue from each treatment group was collected for the analysis of total protein and antioxidant enzyme activities. The samples were gently blotted to remove surface moisture, rapidly frozen in liquid nitrogen to minimize enzymatic degradation, and homogenized in 10 mL of 0.1 mol·L^{-1} phosphate buffer (PBS, pH 7.4). The resulting homogenates were centrifuged using a refrigerated centrifuge (Heraeus Fresco 17, Thermo Fisher Scientific) at 10,000×g for 10min at 4°C, and the supernatants were subsequently preserved at −80°C until biochemical assays were performed.

Text S4. Experimental procedure for microbiome diversity amplicon sequencing

Only one sample per treatment group was used for 16S sequencing. Genomic DNA was extracted from the samples, and the V3-V4 region of the 16S rDNA was amplified using barcoded specific primers. The primer sequences were: 341F: CCTACGGGNGGCWGCAG and 806R: GGACTACHVGGGTATCTAAT. The purified amplification products (i.e., amplicons) were ligated with sequencing adapters to construct sequencing libraries for sequencing on the Illumina platform.

Following sequencing, raw reads were obtained. Due to PCR errors, sequencing errors, etc., a large amount of low-quality or biologically meaningless data (e.g., chimeras) can be generated. Therefore, to ensure the statistical reliability and biological validity of subsequent analyses, strict quality control was performed on the reads through multiple data processing steps including read utilization and tag assembly. Based on the DADA2 software, reads were filtered and corrected, and non-redundant reads and their corresponding abundance information were output. The reads were then assembled into tags, and chimeric tags were removed to obtain tag sequences and abundances for subsequent analysis, namely Amplicon Sequence Variants (ASV) sequences and abundance information. Based on the ASV sequence and abundance data, species annotation, species composition analysis, indicator species analysis, alpha diversity analysis, and community function prediction were carried out. Due to the lack of biological replicates in the microbiome analysis, statistical testing for differences in community structure was not performed.

Text S5. Transcriptome analysisd

Following 28 days of exposure, samples were collected for transcriptome sequencing. The harvested cells were centrifuged at 4000 rpm for 5 min, rinsed with phosphate-buffered saline (PBS), rapidly frozen in liquid nitrogen, and stored for subsequent RNA extraction. Total RNA was extracted, reverse-transcribed into cDNA, and used for library construction, followed by high-throughput sequencing to generate transcriptomic profiles. Differentially expressed genes (DEGs) were determined using the DESeq2 package, applying the thresholds of $|log_2FC| \geq 1$ and FDR < 0.05. GO (Gene Ontology Resource) and KEGG (KEGG: Kyoto Encyclopedia of Genes and Genomes) enrichment analyses were conducted to identify functional pathways and molecular networks.

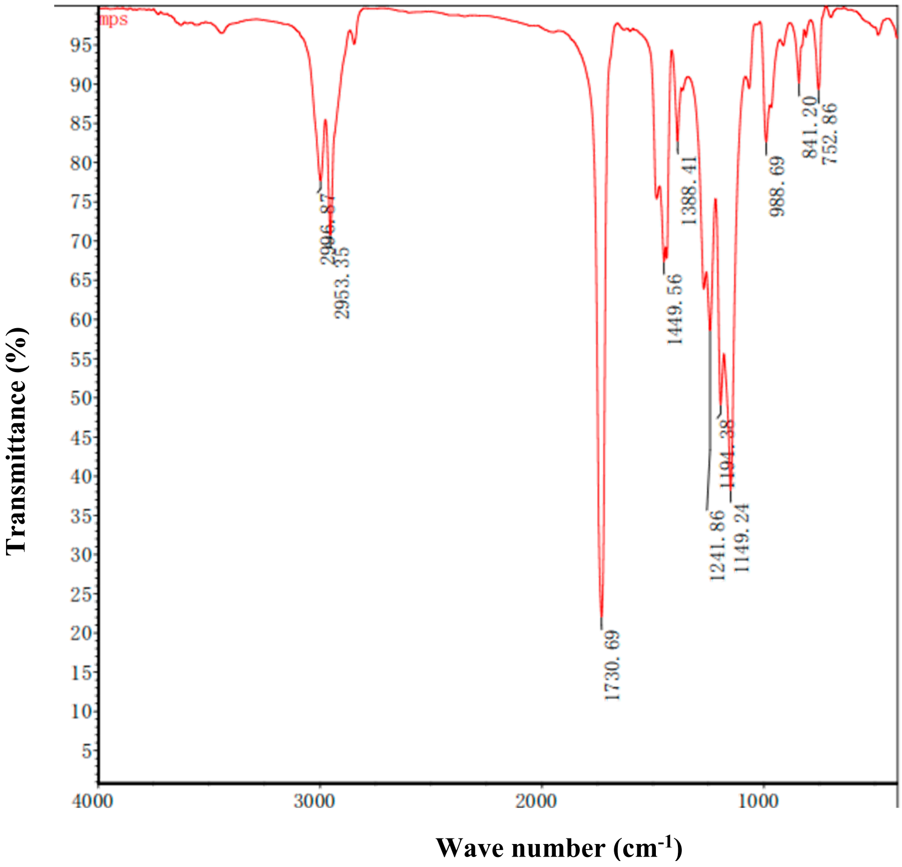

Figure S1. Fourier transform infrared (FTIR) spectrum of polystyrene microplastics. The spectrum shows the characteristic absorption peaks of polystyrene, including aromatic C-H stretching between 3000-2800 cm^{-1}, C=C ring vibrations near 1600 cm^{-1} and 1493 cm^{-1}, and a set of strong absorption bands below 800 cm^{-1}, characteristic of C-H out-of-plane bending in monosubstituted benzene. These characteristic peaks confirm that the chemical composition of this material is polystyrene.

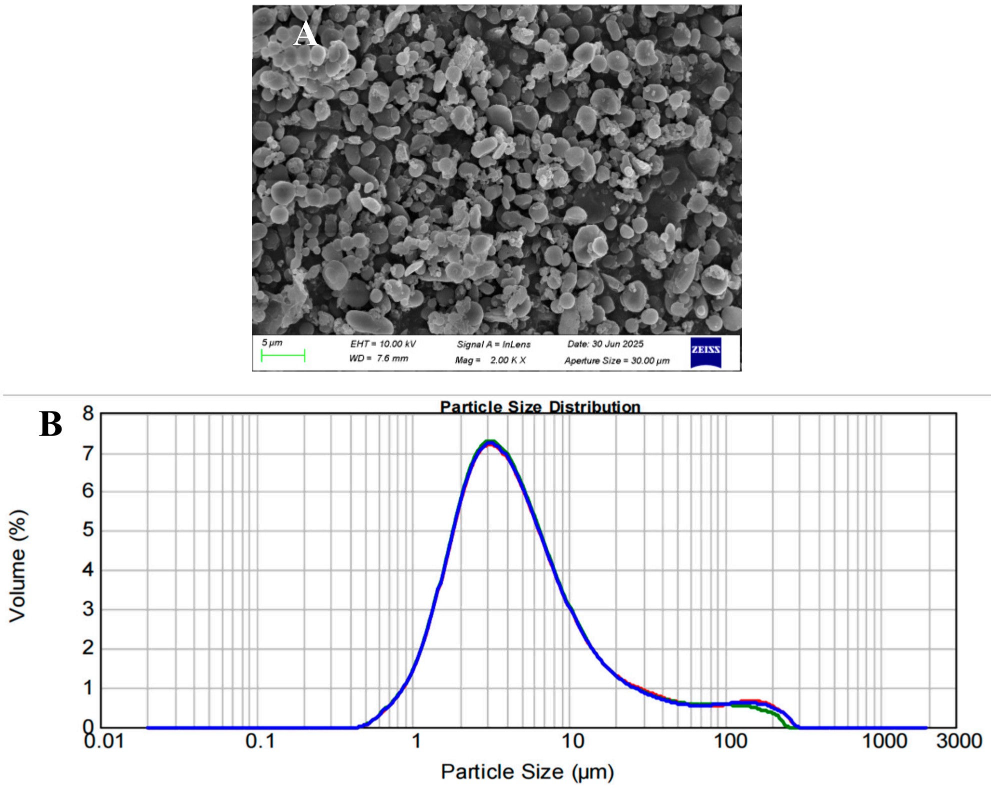

Figure S2. Morphology and size distribution of the polystyrene microplastics(PS). (A) Representative scanning electron microscopy (SEM) image showing the spherical morphology of the PS particles. (B) Particle size distribution of PS particles dispersed in deionized water measured by laser diffraction. The particles exhibited a broad size distribution with a median diameter (d(0.5)) of 4.10 μm. The volume-weighted mean diameter D[4,3] was 13.40 μm, with percentile values of d(0.1) = 1.56 μm and d(0.9) = 23.58 μm. These results confirm that the particles are mainly distributed in the micrometer range and are consistent with the nominal particle size (~3 μm) reported by the manufacturer.

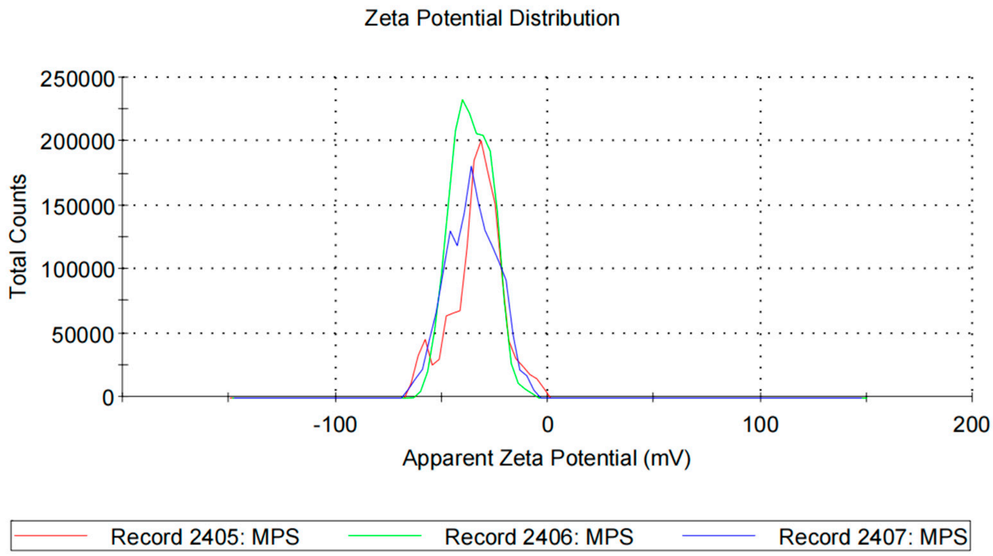

Figure S3. Zeta potential distribution of polystyrene microplastics in deionized water. The distribution exhibits a primary peak at -31.4 mV and a minor secondary peak at approximately -58.5 mV. The intensity-weighted mean zeta potential was -33.2 mV. This strongly negative value (exceeding the absolute threshold of 30 mV) indicates good colloidal stability in the aqueous suspension, which is attributed to strong electrostatic repulsion between particles. The presence of a minor peak suggests a small population of particles with a different surface charge, possibly due to slight variations in size or surface chemistry.

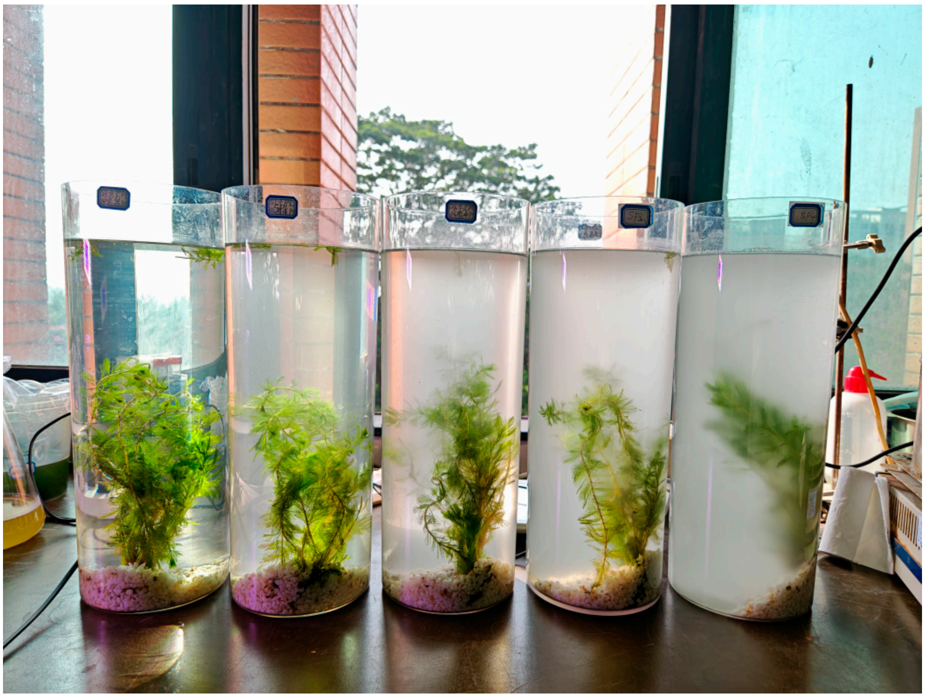

Figure S4. Growth status of *Myriophyllum spicatum* at day 14 of the experiment.

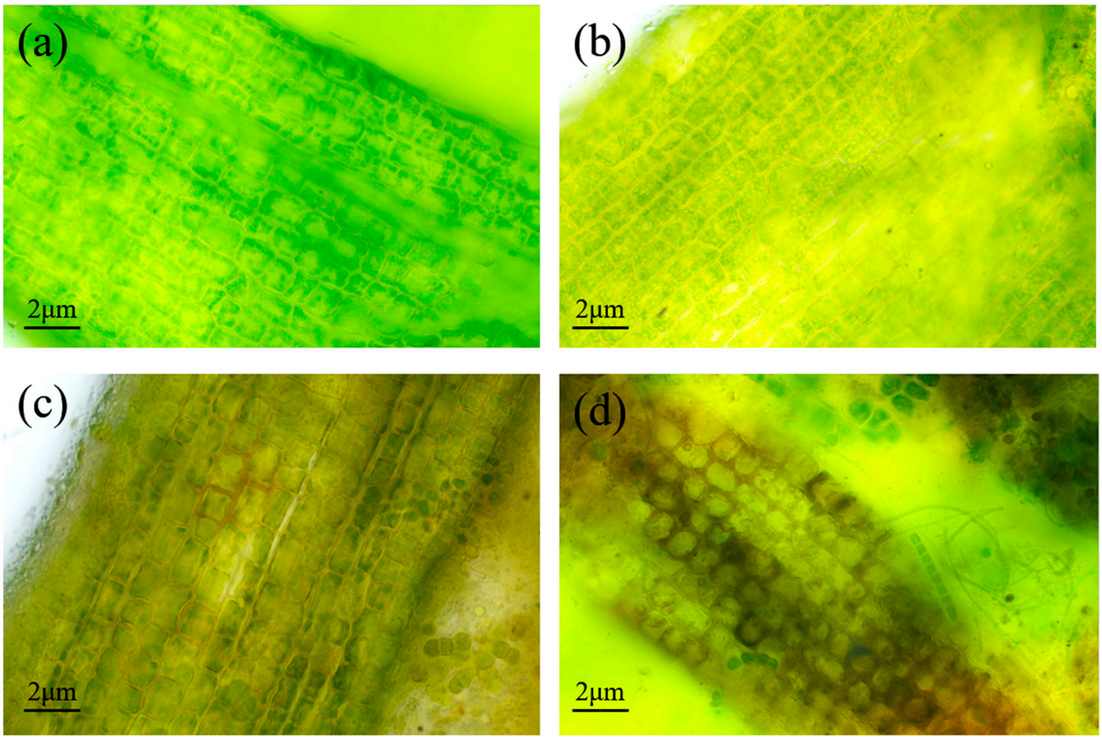

Figure S5. Optical microscopy images of leaf tissues of *M. spicatum* under different microplastic treatments. (a) SP0; (b) SP30; (c) SP60; (d) SP100. Relatively intact cellular morphology was observed in the SP30 group, while higher microplastic concentrations resulted in visible structural disruption, including cell deformation and chloroplast disorganization. Images were captured at a magnification of 40×, scale bar = 2 μm.

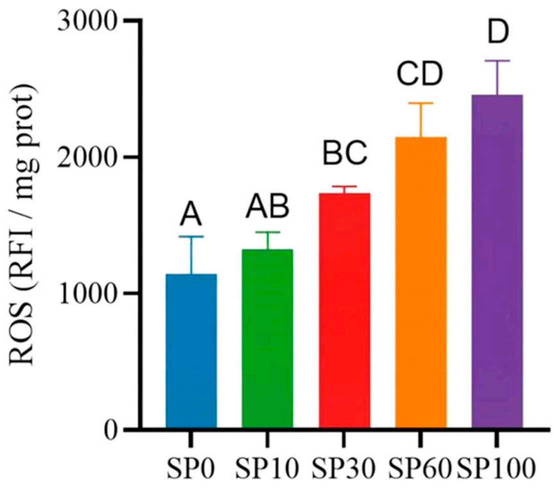

Figure S6. ROS levels under different treatments.

Table S1. The components of sea salt.

#	Component	Concentration (g/100g)
1	$NaCl$	62.26
2	$MgSO_4 \cdot 7H_2O$	14.39
3	$MgCl_2 \cdot 6H_2O$	12.76
4	$CaCl_2$	3.27
5	KCl	1.74
6	Na_2CO_3	0.49
7	$SrCl_2$	0.046
8	KBr	0.026
9	$MnSO_4$	0.00947
10	$KH_2PO_4 \cdot 7H_2O$	0.0071
11	$LiCl$	0.0024
12	$Na_2MoO_4 \cdot 2H_2O$	0.0024
13	$(C_6H_{11}O_7)_2Ca$	0.00059
14	$Al_2(SO_4)_3$	0.00043
15	$CuSO_4$	0.0004
16	$RbCl$	0.00014
17	$ZnSO_4 \cdot 7H_2O$	0.000091
18	KI	0.000085
19	$CoSO_4 \cdot 7H_2O$	0.000047

Table S2. The components of Hoagland medium.

#	Component	Concentration (mg/L dH_2O)
1	KNO_3	1010
2	$Ca(NO_3)_2 \cdot 4H_2O$	944
3	$NH_4H_2PO_4$	115
4	$MgSO_4 \cdot 7H_2O$	493
5	H_3BO_3	2.86
6	$MnSO_4 \cdot H_2O$	2.23
7	$ZnSO_4 \cdot 7H_2O$	0.22
8	$CuSO_4 \cdot 5H_2O$	0.08
9	$NaMoO_4 \cdot 2H_2O$	0.02

Annotation: The pH value is 5.8 ± 6.0 at 25 ± 2°C.

1.7 g of Hoagland medium was heated and dissolved in 1000mL distilled water, and autoclaved at 121 °C for 15 min.

Table S3. Nutrient concentrations of the Pearl River in China.

#	**Parameters**	**Concentration**
1	Salinity	0.86%-0.88%
2	NH_4^+-N	9.12-9.23 mg/L
3	NO_3^--N	2.22-2.30 mg/L
4	NO_2^--N	0.21-0.25 mg/L
5	TP	1.20-1.23 mg/L
6	COD	88-93 mg/L
7	DO	6.50-6.70 mg/L
8	pH	7.5-7.8

Table S4. Summary of the transcriptome sequencing data quality.

Sample	Raw reads (bp)	Clean reads (bp)	Q20 (%)	Q30 (%)	GC content (%)
SP0-1	6031530300	6022336690	99.10%	96.81%	45.55%
SP0-2	6072978600	6063859782	99.21%	97.23%	44.98%
SP0-3	5928942300	5919760555	99.30%	97.56%	45.60%
SPL-1	7142811300	7129687586	99.09%	96.77%	45.43%
SPL-2	7274553900	7262625248	99.22%	97.22%	45.32%
SPL-3	5850161700	5842194119	99.26%	97.41%	45.58%
SPH-1	5403570600	5396820160	99.21%	97.23%	45.70%
SPH-2	6138249000	6127919479	99.25%	97.38%	46.11%
SPH-3	6177008400	6167844341	99.21%	97.25%	45.73%

Annotation:

Q20 and Q30 represent the percentage of bases with quality scores ≥ 20 and 30, respectively.

Q20 (%): the percentage of bases with a correct base identification rate of more than 99% (more than 85%).

Q30 (%): the percentage of bases with a correct base identification rate of more than 99.9% (more than 80%).

GC content (%): The total of G and C bases as a percentage of total bases.

Table S5. Expression levels and statistical significance of DEGs.

Gene name	SP0 vs SPL			SP0 vs SPH		
	**log_2FC**	**FDR**	**Significance**	**log_2FC**	**FDR**	**Significance**
LHCB4.1	12.620602	0.000415	up	15.555328	6.65E-11	up
LHCB5	11.617774	0.00364	up	14.879248	1.26E-09	up
PSBO	6.672874	0.007294	up	9.222373	3.61E-08	up
PSBP	5.118824	0.041521	up	7.287982	8.74E-06	up
RBCS3	4.646219	0.030691	up	6.962885	5.07E-08	up
SGR1	-0.135577	0.9211	no	-1.802604	0.006175	down
SOD1	2.354679	0.036111	up	1.8608	0.081253	no
SOD2	6.622275	0.002677	up	6.633633	0.000236	up
CAT1	10.759888	0.007202	up	13.789228	2.36E-09	up
POD	3.061242	0.154679	no	3.634256	0.000516	up
GSH1	1.804995	0.366911	no	2.797201	0.039757	up
GST2	11.877668	0.028397	up	12.646034	0.000536	up

Table S5. (continued).

Gene name	SP0 vs SPL			SP0 vs SPH		
	log_2FC	FDR	Significance	log_2FC	FDR	Significance
AMT1.1	4.095924	0.001928	up	3.380492	0.003557	up
GLN4	12.026754	0.000173	up	10.699283	0.049011	up
PHT1.10	0.872328	0.236613	no	1.231647	0.02611	up
PHR2	9.066089	0.15963	no	12.403811	6.41E-07	up
GS1.1	9.595568	0.279418	no	12.291746	6.56E-06	up
SPS3	10.27496	0.001837	up	13.621937	8.98E-11	up
ATPB	6.906162	0.002791	up	9.781409	2.10E-09	up
COX12	3.743681	0.004247	up	2.964416	0.012706	up
RPS13	4.274454	0.000483	up	4.215815	1.65E-07	up
RPS11	14.452306	0.000045	up	12.039148	0.002584	up
LOX4	9.083036	0.040227	up	12.468963	5.69E-08	up
PIP2.7	-2.007794	0.348903	no	-3.630882	0.000027	down
LTPG1	-1.497113	0.344269	no	-2.497795	0.000091	down

Table S6. Summary statistics of sequencing data for all samples.

Sample	Number of sequences	Total bases (bp)	Max length (bp)	Min length (bp)	ASV number
SP0-1	46167	22200530	508	252	259
SP0-2	61004	27913421	444	262	245
SP0-3	54599	24743480	432	318	252
SP10-1	57449	28783925	525	253	270
SP10-2	56880	26563367	450	294	217
SP10-3	57203	25935341	511	316	243
SP30-1	44197	23341101	528	232	333
SP30-2	53750	24216560	432	262	230
SP30-3	44146	20138311	431	366	210
SP60-1	37666	17766801	517	245	248
SP60-2	62457	29442793	450	230	274
SP60-3	52046	24283762	432	262	289
SP100-1	51442	24838388	497	219	329
SP100-2	37778	17150688	434	401	171
SP100-3	49386	22451565	432	369	224

Annotation:

Sample: Sample Name

Number of sequences: number of optimized sequencing reads for each sample.

Total bases (bp): total number of bases obtained after quality filtering.

Minimum length (bp): the shortest reads obtained for each sample.

maximum length (bp): the longest reads obtained for each sample.

ASV number: Amplicon Sequence Variant, representing the number of microbial taxa in each sample.

Table S7. Spearman correlation coefficients (r value) between physiological indices and key microbial genus.

**Microbial genus**	**Shoot length**	**Fresh weight**	**Total chl**	**chla/chlb**	**MDA**	**SOD**	**CAT**	**POD**	**GSH**	**GST**	**NH_4^+-N removal rate**	**TP removal rate**
norank_o__Chloroplast	-0.100	-0.164	-0.064	0.510	0.271	0.146	0.096	0.211	0.189	0.246	-0.256	-0.411
unclassified_f__Paracoccaceae	0.168	0.218	0.186	-0.111	-0.193	-0.025	0.050	0.068	0.068	0.039	-0.070	0.013
unclassified_f__Rhizobiaceae	-0.139	-0.143	-0.286	-0.140	0.136	0.296	0.321	0.261	0.321	0.243	0.150	-0.059
*Geitlerinema*_LD9	-0.143	0.136	0.034	0.124	0.004	-0.145	-0.107	-0.064	-0.170	-0.157	-0.385	-0.111
norank_o__SepB-3	-0.525	-0.300	-0.554	0.479	0.461	0.257	0.168	0.350	0.243	0.271	-0.634	-0.495
Fuscovulum	0.331	0.129	0.239	-0.641	-0.558	-0.334	-0.379	-0.420	-0.475	-0.450	0.608	0.674
norank_f__Leptolyngbyaceae	0.300	0.318	0.393	-0.148	-0.368	-0.361	-0.404	-0.361	-0.429	-0.368	0.020	0.306
Flavobacterium	0.054	0.150	0.193	-0.358	-0.150	0.154	0.182	0.050	0.050	0.036	0.095	0.086
unclassified_f__Sphingomonadaceae	0.114	0.004	-0.036	-0.492	-0.300	0.050	0.054	-0.043	-0.082	-0.114	0.411	0.381
Rhizobium	-0.345	-0.225	-0.526	0.063	0.304	0.621	0.592	0.537	0.547	0.431	-0.172	-0.289

Table S8. Spearman correlation coefficients (p value) between physiological indices and key microbial genus.

**Microbial genus**	**Shoot length**	**Fresh weight**	**Total chl**	**chla/chlb**	**MDA**	**SOD**	**CAT**	**POD**	**GSH**	**GST**	**NH_4^+-N removal rate**	**TP removal rate**
norank_o__ Chloroplast	0.724	0.558	0.822	0.054	0.327	0.602	0.734	0.450	0.498	0.375	0.355	0.128
unclassified_f__ Paracoccaceae	0.549	0.434	0.507	0.692	0.490	0.934	0.863	0.812	0.812	0.893	0.805	0.967
unclassified_f__ Rhizobiaceae	0.621	0.611	0.301	0.618	0.630	0.283	0.242	0.347	0.242	0.382	0.591	0.835
*Geitlerinema*_LD9	0.609	0.627	0.906	0.658	0.992	0.604	0.702	0.820	0.543	0.573	0.157	0.692
norank_o__SepB-3	0.047	0.277	0.035	0.072	0.086	0.354	0.549	0.201	0.382	0.327	0.013	0.063
Fuscovulum	0.227	0.645	0.387	0.012	0.033	0.222	0.163	0.120	0.075	0.093	0.018	0.007
norank_f__ Leptolyngbyaceae	0.277	0.248	0.149	0.595	0.178	0.187	0.137	0.187	0.113	0.178	0.946	0.266
Flavobacterium	0.852	0.593	0.490	0.190	0.593	0.584	0.515	0.863	0.863	0.903	0.736	0.760
unclassified_f__Sp hingomonadaceae	0.686	0.995	0.903	0.064	0.277	0.863	0.852	0.883	0.773	0.686	0.128	0.161
Rhizobium	0.206	0.416	0.046	0.823	0.269	0.015	0.022	0.041	0.037	0.110	0.537	0.293

Table S9. Mantel test results showing correlations between key microbial genera and plant physiological traits.

Microbial genus	Statistic	Shoot length	Fresh weight	Total chl	chla/chlb	MDA	SOD	CAT	POD	GSH	GST	NH_4^+-N removal rate	TP removal rate
norank_o__SepB-3	Mantel r	0.207	0.066	0.394	0.227	0.255	0.271	0.066	0.298	0.292	0.180	0.485	0.394
	p value	0.088	0.295	0.003	0.071	0.021	0.039	0.213	0.017	0.015	0.092	0.002	0.003
Fuscovulum	Mantel r	-0.014	-0.194	0.106	0.291	0.177	0.259	0.051	0.123	0.254	0.068	0.428	0.414
	p value	0.499	0.959	0.16	0.04	0.079	0.05	0.28	0.165	0.037	0.31	0.001	0.005
Rhizobium	Mantel r	-0.118	-0.0956	0.167	0.0178	0.0691	0.338	0.245	0.281	0.279	0.290	0.11	0.089
	p value	0.807	0.696	0.073	0.422	0.268	0.02	0.021	0.023	0.021	0.016	0.184	0.237
